# Assessing the reliability of web-based measurements of visual function

**DOI:** 10.3758/s13428-022-02057-2

**Published:** 2023-01-23

**Authors:** Richard J. Leadbeater, Paul McGraw, Timothy Ledgeway

**Affiliations:** Visual Neuroscience Group, School of Psychology, University of Nottingham, Nottingham, NG7 2RD UK

**Keywords:** Vision, Remote vision testing, Web-based testing, Web / lab comparison, Orientation perception, Reliability and agreement, Oblique effect

## Abstract

Many behavioural phenomena have been replicated using web-based experiments, but evaluation of the agreement between objective measures of web- and lab-based performance is required if scientists and clinicians are to reap the benefits of web-based testing. In this study, we investigated the reliability of a task which assesses early visual cortical function by evaluating the well-known ‘oblique effect’ (we are better at seeing horizontal and vertical edges than tilted ones) and the levels of agreement between remote, web-based measures and lab-based measures. Sixty-nine young participants (mean age, 21.8 years) performed temporal and spatial versions of a web-based, two-alternative forced choice (2AFC) orientation-identification task. In each case, orientation-identification thresholds (the minimum orientation difference at which a standard orientation could be reliably distinguished from a rotated comparison) were measured for cardinal (horizontal and vertical) and oblique orientations. Reliability was assessed in a subsample of 18 participants who performed the same tasks under laboratory conditions. Robust oblique effects were found, such that thresholds were substantially lower for cardinal orientations compared to obliques, for both web- and lab-based measures of the temporal and spatial 2AFC tasks. Crucially, web- and lab-based orientation-identification thresholds showed high levels of agreement, demonstrating the suitability of web-based testing for assessments of early visual cortical function. Future studies should assess the reliability of similar web-based tasks in clinical populations to evaluate their adoption into clinical settings, either to screen for visual anomalies or to assess changes in performance associated with progression of disease severity.

## Web-based research

There is a growing body of research showing that behavioural phenomena typically discovered under laboratory conditions can be replicated using online, web-based testing methods (Crump et al., 2013; Sauter et al., 2020; Woods et al., 2015). Some researchers have attempted to demonstrate agreement with lab-based tasks to validate the use of web-based testing, but with mixed success (Germine et al., 2012; Khurana et al., 2021; Q. Li et al., 2020; Wang et al., 2013). Web-based testing is an attractive tool for researchers as larger samples do not necessitate a higher workload, and multiple datasets can be collected simultaneously at the convenience of both participant and experimenter. Online recruitment methods, such as crowdsourcing, allow researchers to recruit larger, more diverse samples than usually found through local recruitment methods (Stewart et al., 2017). For clinical research, web-based testing removes the need for patients to organise and pay for transportation to reach clinical facilities, allowing hard-to-reach populations, such as those with severe visual dysfunction, to conceivably be tested in their home environment.

The potential of web-based testing is appealing, but there are obvious concerns about data quality in the absence of experimental supervision in standardised testing environments. Although online participants self-report higher levels of distraction than those in the lab (Jun et al., 2017), performance on various attentional checks is similar for web- and lab-based studies (Clifford & Jerit, 2014; Jun et al., 2017), even when sustained attention is required over prolonged periods (Gould et al., 2015). High exclusion rates are common in web-based research that recruit participants through crowdsourcing methods due to participant anonymity, lower rates of inconvenience allowance, and a lack of interest in the experiment (Thomas & Clifford, 2017; Zhou & Fishbach, 2016).

## Applications of web-based methods

Currently, the majority of research evaluating the data quality of web-based methods are interested in the replication of task-based effects (Crump et al., 2013; Sauter et al., 2020). Comparable data quality has been demonstrated for a variety of time-insensitive tasks, such as surveys (Bartneck et al., 2015; Buhrmester et al., 2011; Casler et al., 2013; Clifford & Jerit, 2014), problem solving (Dandurand et al., 2008), and decision making (Paolacci et al., 2010). The data quality of time-sensitive tasks, such as those measuring reaction times, is more susceptible to lapses in attention or variability in stimulus and response timings. For example, there is poor agreement between web- and lab-based response timings with discrepancies over ~ 80 ms (Semmelmann & Weigelt, 2017). Reaction-time research on the web thus focuses on replicating task-based effects (e.g. Stroop, flanker, visual search, attentional blink) by comparing performance between experimental conditions or between groups (Armitage & Eerola, 2020; Crump et al., 2013; de Leeuw & Motz, 2016; Hilbig, 2016; Kim et al., 2019; Vancleef et al., 2017). Measures of relative performance control for inter-individual variability in group analyses, but critically say nothing about the agreement between web- and lab-based measures (Q. Li et al., 2020; Vancleef et al., 2017). The assessment of agreement between web- and lab-based methods requires objective evaluations of task performance (e.g. thresholds, error rates). Therefore, within-subjects designs and forced-choice procedures are well suited for investigations of reliability (Vancleef et al., 2017; Wang et al., 2013).

Special considerations are required for web-based vision experiments performed on personal computers with visual display equipment outside of the experimenter’s control. Tasks must be robust to variability in hardware, operating systems, and visual display settings such as screen resolution or luminance scaling. This makes some measures of visual function, such as contrast sensitivity and visual acuity, more challenging for web-based testing. Indeed, web-based measures of contrast sensitivity may yield markedly larger thresholds (Sasaki & Yamada, 2019) than expected under conventional laboratory conditions (Campbell & Kulikowski, 1966; Heeley & Timney, 1988; Westheimer & Beard, 1998). One solution is to provide participants with devices which are specifically calibrated for a given visual task (Khurana et al., 2021; Wang et al., 2013). For example, Khurana et al. (2021) measured patients’ visual acuity on calibrated hand-held devices and reported a strong relationship between home-based measurements and clinic-based measurements. However, crucially their analyses of agreement were not shown. It is therefore unclear whether there were any one-to-one, or systematic differences between the measures (Armstrong, 2019; McAlinden et al., 2011).

## Reliability of web-based methods

When quantifying the level of agreement between measures, it is vital that appropriate statistical tests are utilised. For example, the intraclass correlation coefficient (ICC) quantifies the reliability between measures and can account for one-to-one differences (Koo & Li, 2016), and Bland–Altman analyses assess the level of agreement between measures and can quantify and control for any systematic differences (Bland & Altman, 1986, 1999). Demonstrating agreement between web- and lab-based measurements in visual tasks is highly important for any potential clinical applications of web-based testing, especially where individual performance is associated with pathological structural changes (Bedell et al., 2009; Fu et al., 2017; Ogata et al., 2019; Wang et al., 2013).

In the present study, we explore the reliability of psychophysical (behavioural) measurements of orientation-identification thresholds, obtained on temporal and spatial versions of a web-based, two-alternative forced choice task (2AFC). We aimed to reproduce the well-known oblique effect, whereby visual performance is poorer for oblique orientations compared with cardinals (horizontal and vertical), which has previously been reported for 2AFC orientation-discrimination tasks (Campbell et al., 1966; Heeley & Timney, 1988; Westheimer, 2003). We explore the agreement between web- and lab-based measures on both the temporal and spatial 2AFC tasks and examine the suitability of assessing visual function by manipulating stimulus orientation in web-based tasks.

## Method

### Participants

Eighty-one participants, made up of a mixture of students and staff at the University of Nottingham and members of the general public, took part in the web-based, orientation-identification experiment. Participants were recruited via a variety of methods, including posters, internal mailing lists, and an external volunteer participation pool. Twelve out of 81 participants were excluded from subsequent data analyses as they failed to reach threshold performance levels of responding (i.e. at least 75% correct) in one or more conditions. Of the remaining 69 participants, 59 were female and ten were male, with a mean (SD) age of 21.8 (6.02) years.

To assess the reliability of web-based measurements, a validation-subsample also performed the experiment under laboratory conditions. The sample size was planned assuming an ‘acceptable reliability’ (intraclass correlation coefficient, ICC) of 0.50 and an ‘expected reliability’ (ICC) of 0.75, which respectively correspond to qualitative boundaries of ‘moderate’ and ‘good’ reliability scores (Koo & Li, 2016). With eight measurements per participant on each of the temporal and spatial 2AFC tasks, a minimum sample of 15 participants was required (Bujang & Baharum, 2017). To accommodate for potential participant attrition, 20 participants were recruited in total, with two participants excluded from subsequent data analyses as they failed to reach threshold performance levels of responding. Of the remaining 18 participants, 13 were female and five were male with a mean (SD) age of 27.9 (8.97) years. Counterbalancing of the order of testing meant that nine of 18 participants completed the web-experiment first followed by the lab experiment, and the remaining nine completed the lab-experiment first followed by the web experiment.

Participants were not screened for ocular pathology or visual dysfunction, but were instructed to wear appropriate refractive correction, if required. The experimental procedures complied with the Declaration of Helsinki and were approved by the local ethics committee (School of Psychology, University of Nottingham, UK).

### Apparatus

The web-based experiment was designed in PsychoPy and run through Pavlovia, a web-based testing platform (Peirce et al., 2019), with custom JavaScript code. Participants used their own devices and web-browsers to run the experiment. They were asked to sit in a dimly lit room with no visual distractions (e.g. flashing lights), set their screen brightness to its maximum, turn off any blue-light filters, and adjust the volume of their device to ensure that it was clearly audible. The particular devices used were not recorded. Prior to each run, participants were instructed to maintain a fixed viewing distance of 60 cm (e.g. by cutting a piece of string to length and adjusting their seating position to ensure that the ends of the string, when taut, just touched both the display and the bridge of the nose) and input the width, in cm, of the visible (active) portion of their visual display. Along with this information, we also retrieved the spatial resolution of each participant’s full-screen browser window, making it possible to standardise the size and spatial frequency of stimuli across different visual displays.

Visual stimuli were Gabor patterns composed of oriented 1 cyc/° sinusoidal luminance gratings presented within a Gaussian window (space-constant 1.33 °, truncated at ± 4 °) and at a contrast that was set to be 40% of the maximum available from the visual display. To minimise luminance artefacts, the sinusoidal waveform was always presented in sine phase with respect to the centre of the Gaussian envelope. Whether the grating was a positive sine wave or negative sine wave was randomised on each presentation for each grating. On the temporal 2AFC task, two stimuli were presented sequentially in a central location. On the spatial 2AFC task, two stimuli were simultaneously presented to the left or right of a central fixation spot. The remainder of the screen was a homogenous “mid-grey” field.

### Design

Orientation-identification thresholds were determined for both the temporal and spatial 2AFC tasks, using the method of constant stimuli. There were four orientation conditions which set the value of the standard orientation (either 0, 90, –45, or +45°) which was constant for each run of trials. On each trial the comparison orientation, which always differed from that of the standard, was chosen at random from one of a predetermined set of seven levels based on pilot studies. Our pilot data revealed a substantial impairment in performance for oblique orientations relative to cardinals (horizontal and vertical), therefore, the orientation-offsets between the standard and comparison were distinct for cardinals (0.71, 1.43, 2.14, 2.86, 3.57, 4.29, and 5.00 °) and obliques (3.57, 7.14, 10.70, 14.29, 17.80, 21.42, and 25.00 °). Each run comprised of ten repeats of the seven orientation-offset levels, the sign of which (i.e. whether the comparison was clockwise or anti-clockwise relative to the standard) was randomised on each presentation. The proportion of correct responses at each orientation-offset level was recorded upon completion of each run. Participants were required to complete five runs (a total of 350 trials) on each orientation condition for each task. Each orientation condition on the temporal and spatial 2AFC tasks took approximately 15 min to complete. All runs were completed in a pseudorandom fashion.

### Procedure

Prior to formal data collection, all participants were directed to practice versions of the temporal and spatial 2AFC tasks, where seven trials were performed to familiarise them with the experiment. In the formal web experiment, participants initiated each run by clicking the relevant URL for each task. At the beginning of each run, participants were first presented with a dialogue box which prompted them to enter their participant identifier, monitor width (cm), and select their chosen standard orientation condition. The participants were also reminded to maintain their viewing distance at 60 cm. Once this information was submitted, participants’ browser windows were forced to full screen and written instructions explained that they were required to identify which of two stimuli was presented at the standard orientation. An example image of a Gabor at the chosen standard orientation was also presented. Participants started the experiment when ready and the example target orientation was removed from the screen.

**Fig. 1:**
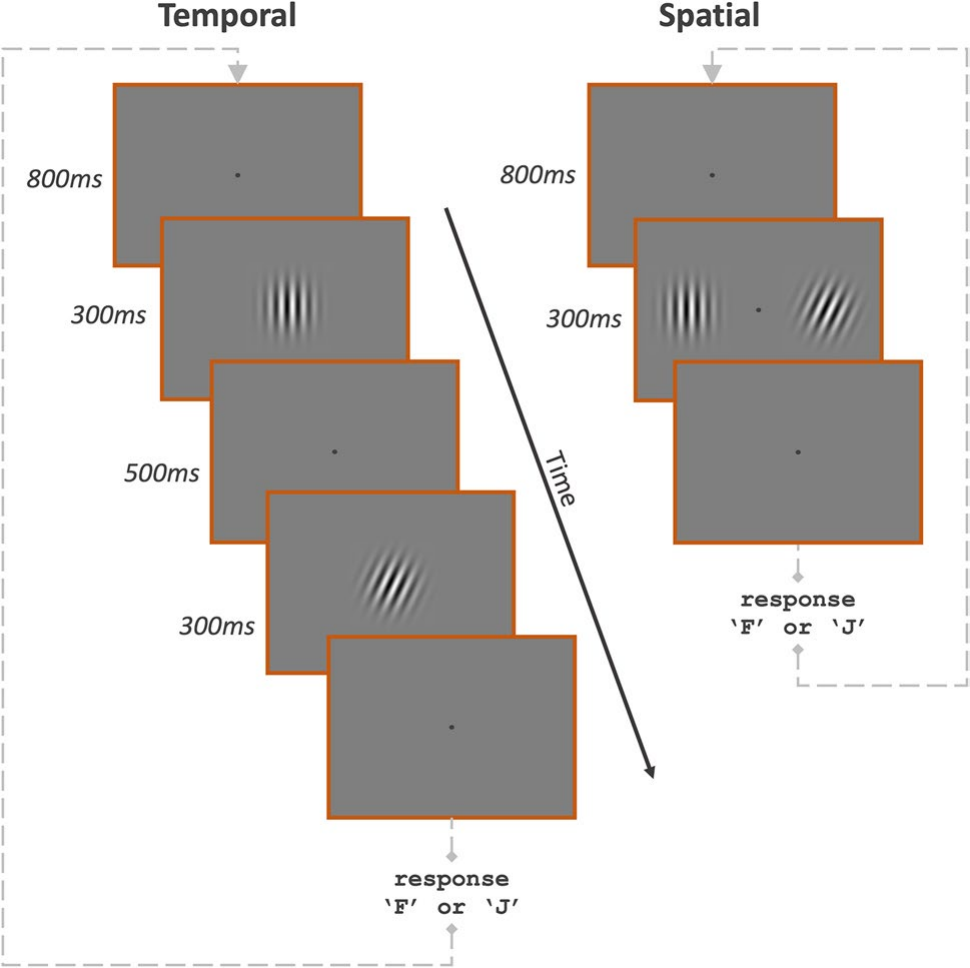
The time-course of the procedure in the temporal (*left*) and spatial (*right*) 2AFC orientation-identification tasks. In the temporal task, participants responded with the ‘F’ or ‘J’ key if the standard orientation appeared in the first or second interval, respectively. In the spatial task, participants responded with the ‘F’ or ‘J’ key if the standard orientation appeared to the left or right of fixation, respectively

On the temporal 2AFC task (Fig. 1), the standard and comparison were presented sequentially for 300 ms at the centre of the screen, with a 500 ms inter-stimulus interval. Stimulus intervals were marked by distinct audible tones. Participants judged in which stimulus-interval the target orientation appeared using the ‘F’ and ‘J’ keys to refer to the first and second intervals, respectively. The order of presentation was randomised.

On the spatial 2AFC task (Fig. 1), participants were twice reminded to maintain their fixation on a central spot throughout each run. Standard and comparison stimuli were presented simultaneously for 300 ms, with the centre of each Gabor being either 8° to the left or right of central fixation. Participants judged in which spatial location the target orientation appeared using the ‘F’ and ‘J’ keys to refer to the left and right locations, respectively. The order of locations was randomised. To aid central fixation, the fixation spot was ever-present except for a brief 100 ms period after each response to indicate that a valid keyboard response had been collected. In both tasks, a response signalled the initiation of the next trial after an 800 ms delay. No response feedback was given.

### Lab-based validation of web-based results

The lab experiment was analogous to the web experiment but took place under carefully controlled conditions at the University of Nottingham campus. Stimuli were generated using an Apple Macintosh computer and presented on a 20-inch CRT monitor (iiyama Vision Master Pro 514) with a refresh rate of 60 Hz, display resolution of 1152 x 870 pixels, and a maximum luminance of 51.63 cd/m^2^. Monitor output was carefully linearised through photometric gamma correction (Minolta LS110 Luminance Meter), and a widely used dithering algorithm provided precise control of luminance resolution (Allard & Faubert, 2008). Testing was conducted in a darkened, quiet room and the viewing distance was fixed at 60 cm by means of a chinrest. Stimulus generation, presentation, and response collection were achieved using PsychoPy (Peirce et al., 2019). Procedural details were identical to those described previously, for the web-based experiment.

### Data analyses

**Fig. 2:**
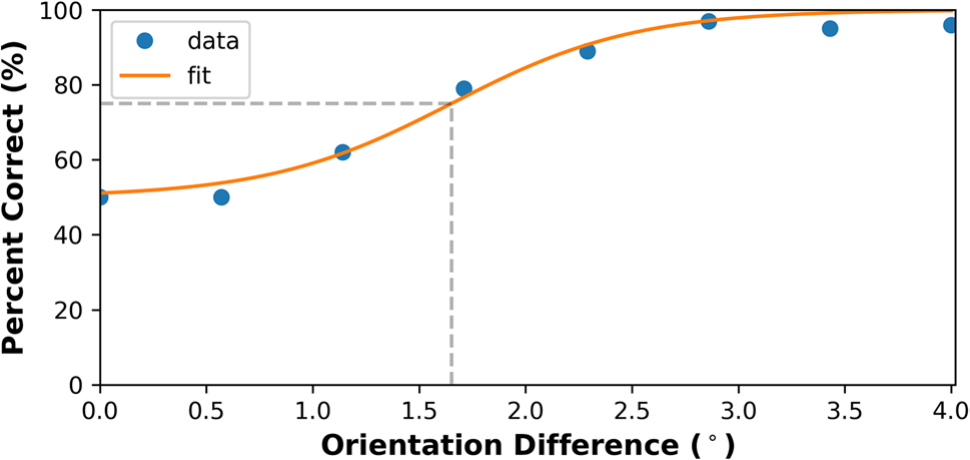
Example of a logistic curve (*orange line*) fit to the psychometric function (*data points*). The threshold (*grey dotted line*) is defined as the orientation difference on the curve corresponding to 75 % correct

All data analyses were carried out using Python. For the temporal and spatial 2AFC tasks, for each participant, the proportion correct for each level of orientation offset was collated across all runs for each orientation condition. These values were used to generate psychometric functions for each condition and a logistic curve was fit to the data using a least-squares method of parameter optimisation to obtain the identification threshold (i.e. the orientation offset on the curve producing 75% correct responses) and the standard error of its estimate (Fig. 2). The logistic function used in the curve-fitting routine had two free parameters, the slope and mid-point of the curve, and was scaled so the minimum value of the curve was at 50% correct performance (Simpson, 1988):

1 $$y=50+50/\left(1+{\textrm{e}}^{\left(x-a\right)/b}\right)$$

where *a* represents the mid-point of the curve, which was taken as the threshold, and *b* represents its slope.

To establish the presence of an oblique effect at the group level in the initial web-based experiment, the mean thresholds for each task (temporal and spatial) and standard orientation were subjected to two-way repeated measures ANOVA and, where appropriate, *t* tests with Bonferroni correction. Effect size is reported as Cohen’s *d* for *t* tests, and (partial) η^2^ for ANOVA.

Reliability was investigated by calculating the intraclass correlation coefficient (ICC) for the temporal and spatial 2AFC tasks. ICC estimates and their 95 % confidence intervals (CIs) were calculated based on single-rater absolute agreement in a two-way random effects model (Koo & Li, 2016; McGraw & Wong, 1996) or ICC_(2,1)_ (Shrout & Fleiss, 1979). Bland–Altman analyses (Bland & Altman, 1986, 1999) of the temporal and the spatial 2AFC tasks were also conducted to investigate the degree of agreement between corresponding orientation-identification thresholds measured in the web- and lab-based versions of the experiment.

## Results

### Web-based orientation-identification experiment

**Fig. 3:**
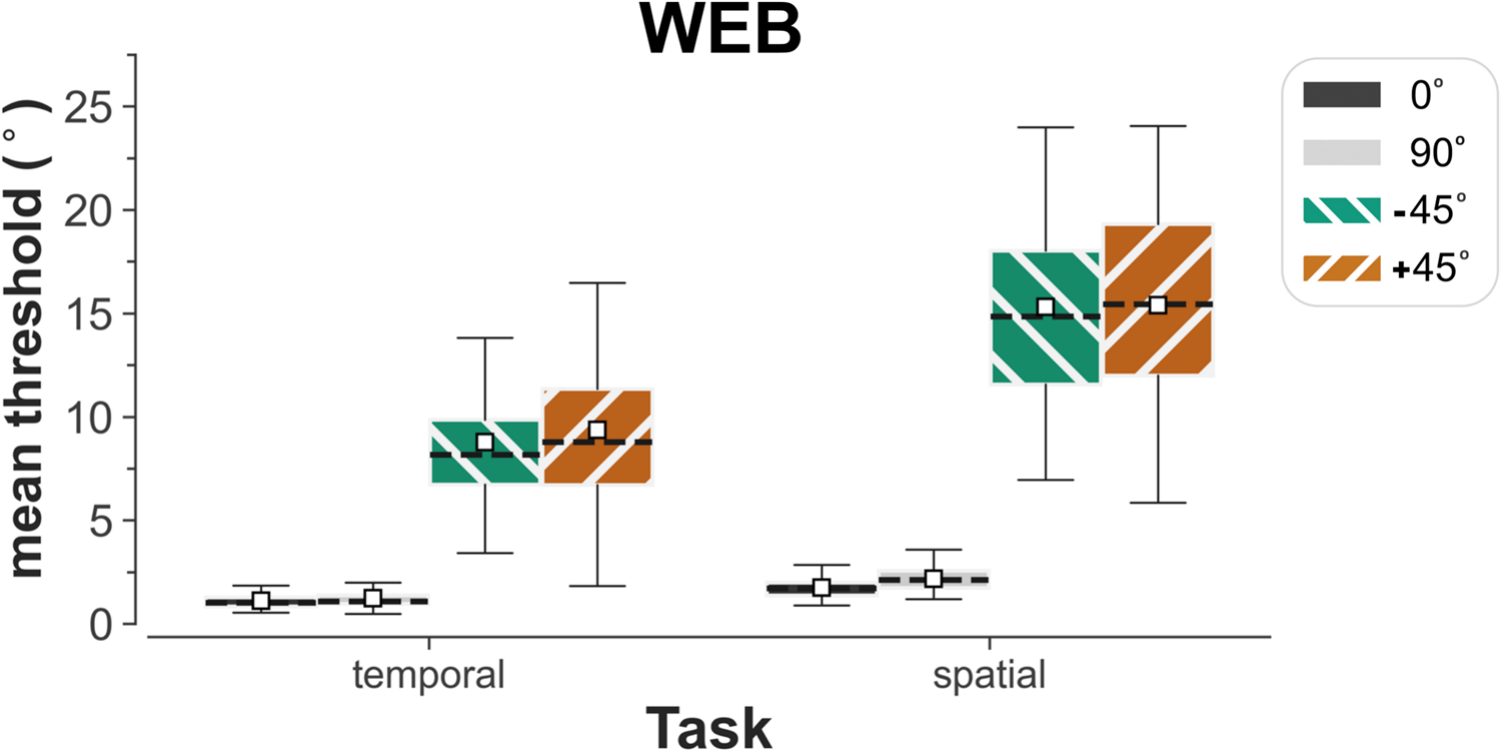
Boxplot showing the means (*white squares*), medians (*dotted line*), and interquartile ranges of thresholds for all participants who took part in the web-based orientation-identification experiment (*n* = 69)

In the web-based experiment, large oblique effects were found in both the temporal and spatial 2AFC tasks (*n* = 69), with substantially lower mean orientation-identification thresholds for horizontal (0 °) and vertical (90 °) orientations relative to obliques (–45 and +45 °) (Fig. 3). In comparison to cardinal orientations (0 and 90 °), there was relatively large variation in the recorded thresholds for obliques. For all tested orientations, mean thresholds were lower on the temporal 2AFC task than on the spatial 2AFC task.

To investigate if the observed differences in performance were significant at the group level in the web-based experiment, the measured thresholds were subjected to two-way (2 x 4), repeated measures ANOVA with the factors of task (temporal 2AFC and spatial 2AFC) and standard orientation (0, 90, –45 and +45 °). Mauchly's test indicated that sphericity had been violated for the main effect of orientation (x^2^(5) = 302.18, *p* < 0.001) and the interaction between orientation and task (x^2^(5) = 200.81, *p* < 0.001). In each case, degrees of freedom were corrected using Greenhouse–Geisser estimates of sphericity (ε = 0.43 and 0.67, respectively). The ANOVA revealed significant main effects of both task (F(1,68) = 455.03, *p* < 0.001, η_p_^2^ = 0.87) and standard orientation (F(1.29, 87.63) = 694.11, *p* < 0.001, η_p_^2^ = 0.91) and a significant interaction between the two factors (F(2.01, 136.58) = 166.63, *p* < 0.001, η_p_^2^ = 0.71). The interaction was investigated by conducting pairwise comparisons (*t* tests) with Bonferroni correction between the mean thresholds for each condition. This revealed that thresholds for the temporal 2AFC task were significantly lower than those for the spatial 2AFC task, for all corresponding orientation conditions (at least t(68) = –11.31, *p* < 0.001, Cohen’s *d* = –1.19). Crucially, thresholds for each cardinal orientation were significantly lower (at least t(68) = –21.99, *p* < 0.001, Cohen’s *d* = –3.35) than for each oblique orientation on the same task. Furthermore, the threshold for identifying the horizontal (0 °) standard orientation showed a modest advantage over that for the vertical (90 °) standard, but only for the spatial 2AFC task (t(68) = –8.76, *p* < 0.001, Cohen’s *d* = –0.67). No other differences reached significance.

### Lab-based validation of web-based measurements

**Fig. 4:**
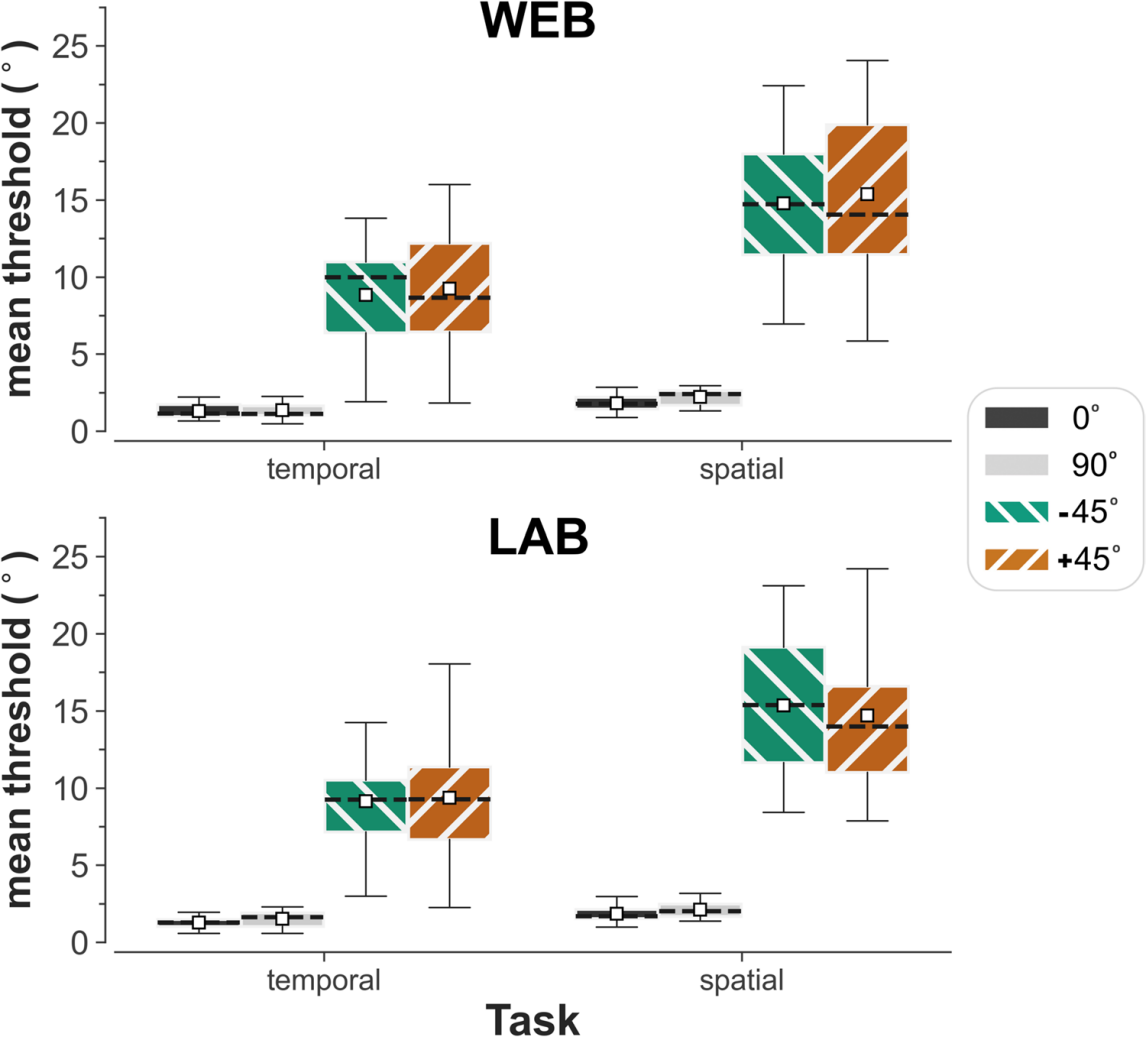
Boxplot showing the means (*white squares*), medians (*dotted line*), and interquartile ranges of thresholds for a validation-subsample (*n* = 18) who performed both the web- (*top*) and lab-based (*bottom*) versions of the orientation-identification experiment

**Fig. 5:**
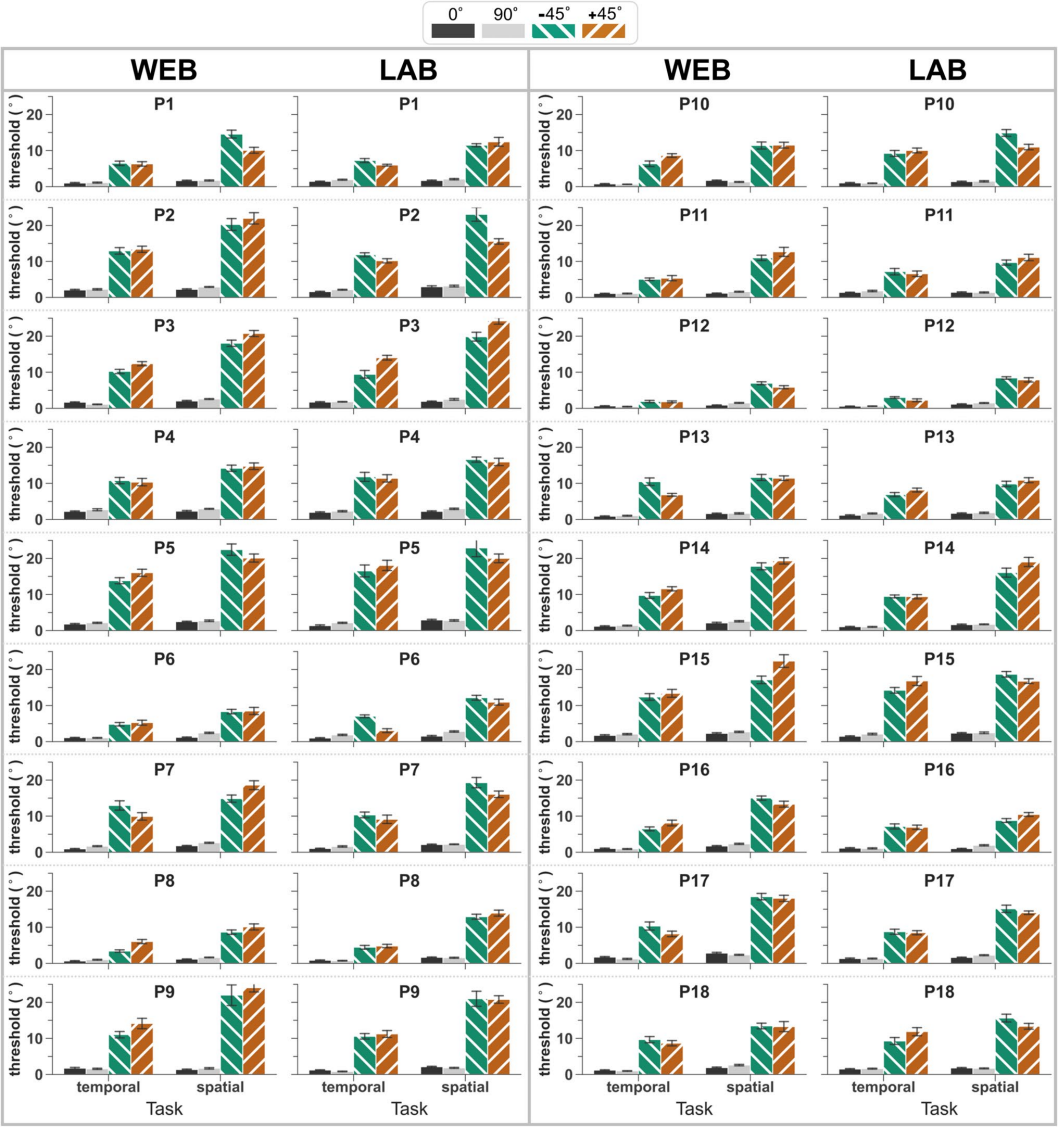
Comparison of web- and lab-based orientation-identification thresholds for each participant in the validation-subsample (*n* = 18). *Error bars* represent the standard error of the threshold estimate

A validation-subsample of participants (*n* = 18) performed both the web-based and lab-based versions of the experiment. Box plots depicting the consistency of thresholds for the subsample as a whole are displayed in Fig. 4, and thresholds for each individual participant are shown in Fig. 5. In both the temporal and spatial versions of the task, orientation-identification thresholds were substantially lower for cardinal orientations relative to obliques for every one of the 18 participants (Fig. 5). Again, there was considerable variation in the thresholds for obliques, and mean thresholds were lower on the temporal 2AFC task than on the spatial 2AFC task for all tested orientations.

### Evaluating agreement

**Fig. 6:**
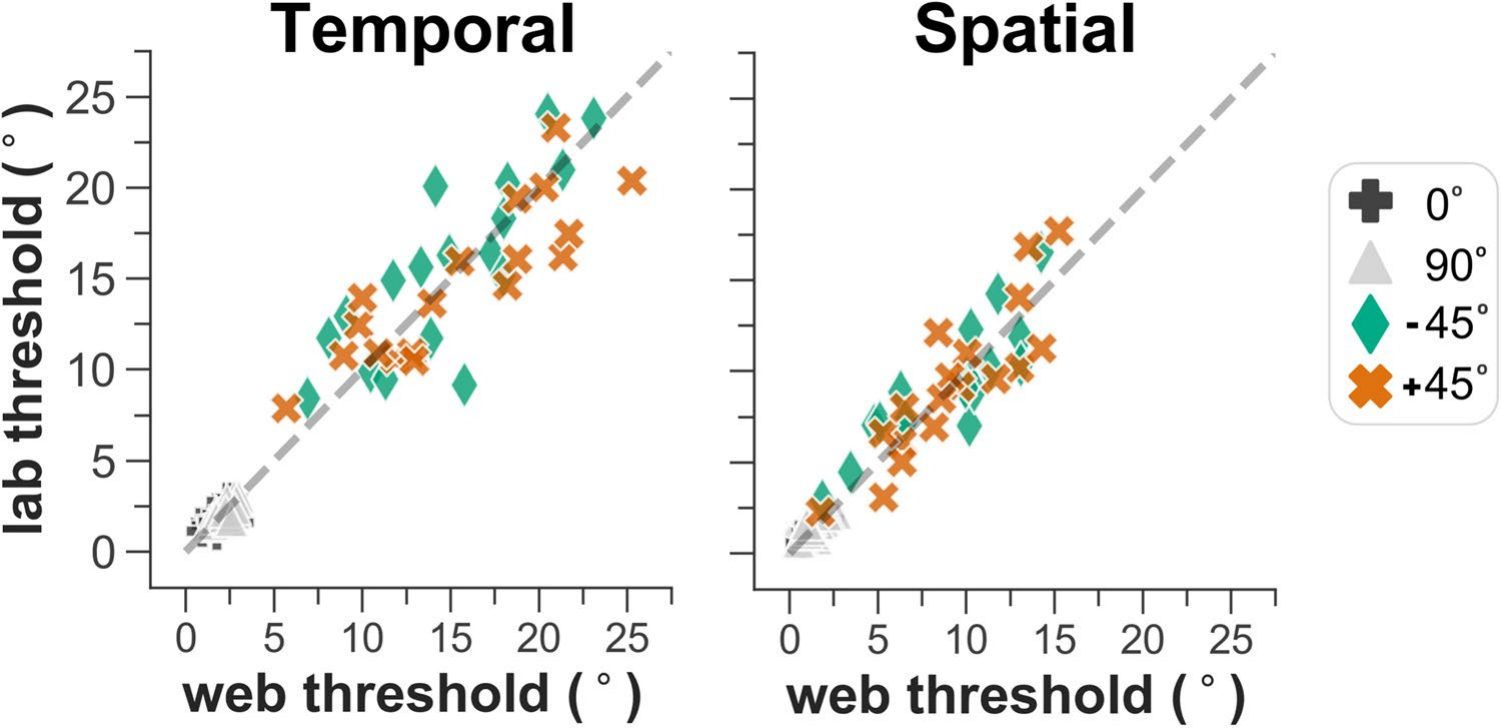
For each participant in the validation-subsample (*n* = 18), thresholds from the lab-based experiment (*y*-axis) are plotted against corresponding thresholds for the web-based experiment (*x*-axis) for both temporal and spatial 2AFC tasks. The line of equality (*dashed line*) is displayed as a reference

Scatterplots demonstrating the overall relationship between the orientation-identification thresholds measured in the web- and lab-based experiments are shown separately for the temporal and spatial 2AFC tasks in Fig. 6. Reliability was quantified by

intraclass correlation coefficient (ICC) estimates and their 95 % confidence intervals. Good-to-excellent reliability was found for the temporal 2AFC task with an ICC (95 % CI) of 0.92 (0.80, 0.97), and the spatial 2AFC task showed moderate-to-good reliability with an ICC (95 % CI) of 0.88 (0.7, 0.94).

**Fig. 7:**
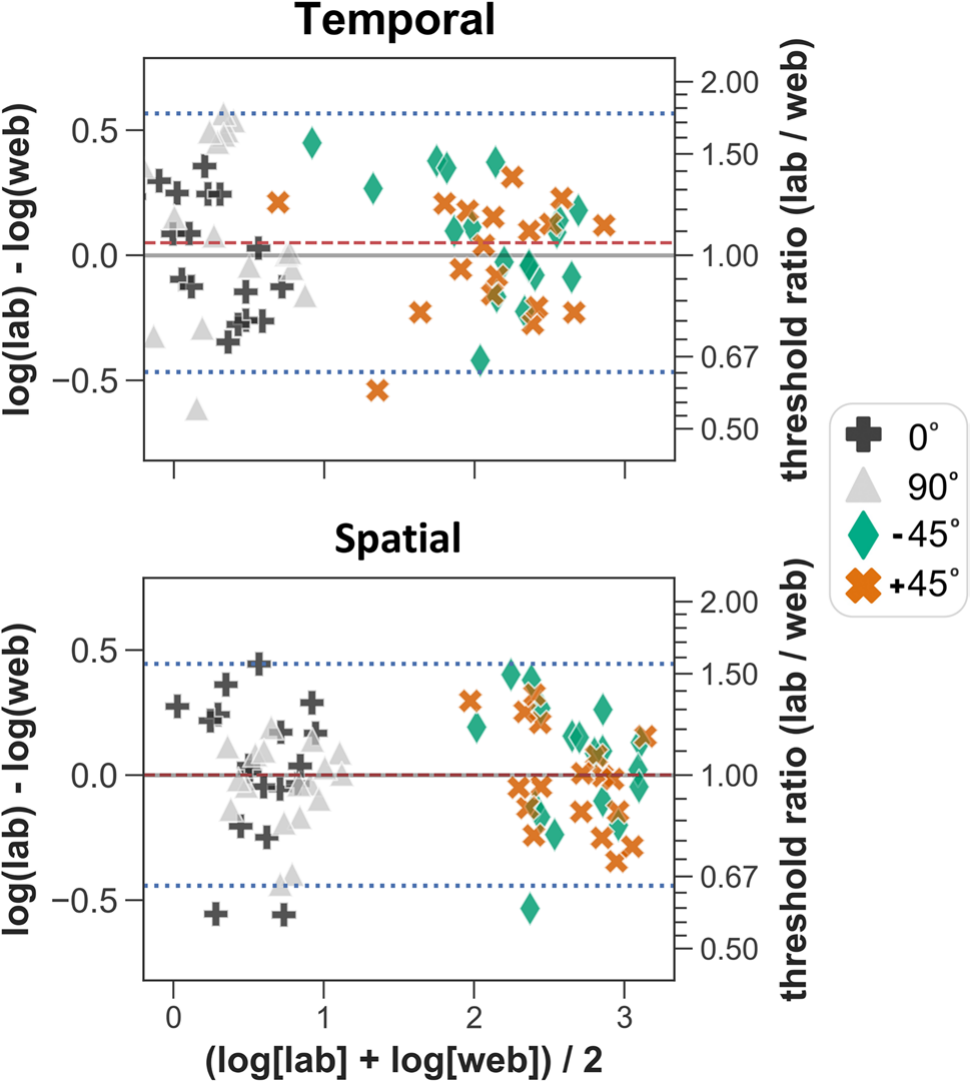
Bland–Altman plots on log-transformed data for the spatial and temporal 2-AFC tasks. For each participant, the difference between lab and web log-thresholds are plotted against the mean log-threshold. The mean difference (*red dashed line*) is shown with the line of equality (*grey solid line*), along with the limits of agreement (*blue dotted lines*). The ratio scale (*right y-axis*) allows visualisation of the magnitude of difference between lab and web thresholds

Agreement between web- and lab-based measurements was evaluated by Bland–Altman analyses (Fig. 7). Due to a proportional relationship between variance and threshold magnitude, Bland–Altman analyses were performed on log-transformed thresholds and the limits of agreement were calculated on a log-scale (Bland & Altman, 1999). The log-transformed data were confirmed not to violate the assumption of normality by Shapiro–Wilk tests. Calculating the antilog of the differences between the log-transformed data enabled differences between web- and lab-based measurements to be examined on a ratio scale, with values under 1 indicating larger thresholds in the web condition, and values over 1 indicating larger thresholds in the lab condition.

The mean difference score between log-transformed thresholds is a measure of systematic bias between web- and lab-based measurements, and the antilog of this gives the mean ratio score. The mean ratio scores were 1.05 and 1.00 on the temporal and spatial 2AFC tasks, respectively, demonstrating the consistency of web- and lab-based threshold measurements in the orientation-identification task. The limits of agreement indicate where the majority (mean difference ± 1 SD * 1.96) of difference scores will lie, and these ranged from 0.63 to 1.76 on the temporal 2AFC task, and from 0.64 to 1.56 on the spatial 2AFC task. The median ‘absolute ratio’ score, calculated from the absolute differences of the log-transformed data, is used here as a metric of typical measurement error between web- and lab-based thresholds. The median ‘absolute ratio’ scores (interquartile range, IQR) were 1.23 (0.25) and 1.16 (0.23) on the temporal and spatial 2AFC tasks, respectively.

To check whether measures of agreement were influenced by potential improvements in performance between experimental sessions (e.g. due to learning effects), threshold differences were examined between participants’ first and second experiments. A mean ratio score of 1.02 (almost identical to the line of equality) was found, indicating that there was little or no influence of learning between experimental sessions on the conducted reliability measures.

## Discussion

### Assessing the reliability of web-based methods

We found robust oblique effects in temporal and spatial versions of a web-based, two-alternative forced choice (2AFC) orientation-identification task, with orientation-identification thresholds for oblique orientations being substantially larger than for cardinals (horizontal and vertical). Similar results were obtained when a validation-subsample of 18 participants completed the same orientation-identification task on the web and under controlled laboratory conditions (e.g. fixed viewing distance, chinrest and headrest, carefully calibrated monitor). This demonstrates the consistency of the task at uncovering the oblique effect regardless of differences in testing environment or inter-individual variability. Furthermore, in our validation-subsample, we found high levels of agreement between web- and lab-based orientation-identification thresholds for both the temporal and spatial 2AFC tasks. These results support the use of web-based methods to assess differences between groups or experimental conditions in measures of early visual cortical function, particularly for tasks which are robust to variability in screen resolution and luminance scaling, such as orientation-discrimination (Shepherd, 2020), visual crowding (Q. Li et al., 2020), global orientation-identification (Bedell et al., 2009; Fu et al., 2017), global motion discrimination (Shepherd, 2020), and shape discrimination (Wang et al., 2002; Wang et al., 2013). Nonetheless, caution should be exercised when generalising results to other web-based experimental tasks.

### Potential applications of web-based methods

Reliable web-based methods could improve the time and resource efficiency of clinical testing by increasing the frequency of assessments without increasing organisational demands for the clinician or patient. Common barriers that can prevent patients from receiving treatment include cost, independent travel, and time commitments (Hartnett, 2005; Javitt & Aiello, 1996; Varano et al., 2015). Web-based methods would be especially useful in cases where performance on a psychophysical task is tracked over time, such as assessments of disease progression or treatment outcomes (Fu et al., 2017; Ogata et al., 2019; Wang et al., 2013), or monitoring of cyclical disorders such as migraine (Shepherd, 2020). However, agreement between web- and lab-based measurements should also be demonstrated in clinical populations before developing web-based tasks for clinical settings. It is reasonable to assume that certain collectives of participants (e.g. clinical populations, older people) interact with technology in a different manner to our sample of healthy, mainly younger participants.

Patients with early, age-related macular degeneration (ARMD) often experience perceptual distortions, due to inhomogeneous structural changes in the retina (Dilks et al., 2014; Ehrlich et al., 2008; Taylor et al., 2018), which can be quantified in tasks that require global integration of visual information over a large spatial extent. Performance on a global ‘orientation-discrimination’ task (Bedell et al., 2009), where observers identify which of two stimuli (groups of short lines) is presented vertically, is associated with the active inflammation status in patients with macular degeneration, and is closely related to the severity of structural changes due to accumulated retinal fluid (Fu et al., 2017). This task is essentially a global version of the temporal 2AFC orientation-identification task presented in this paper, where agreement between web- and lab-based measures was demonstrated. Before such tasks are translated for web-based research in clinical settings, robust and valid assessments of reliability should be made with patient populations.

Glaucoma typically produces a progressive visual loss that disproportionately impacts the peripheral visual field, and the extent of neuropathy has been associated with the magnitude of visual crowding effects measured behaviourally (Ogata et al., 2019). Visual crowding is a phenomenon where features that are clearly identifiable when shown in isolation become difficult to identify when presented together. Ogata et al. (2019) found that glaucomatous eyes showed significantly stronger visual crowding effects, and that this was associated with structural measurements of retinal nerve fibre layer thickness. Q. Li et al. (2020) reported a web-based visual crowding task that successfully differentiated performance levels between dyslexic and non-dyslexic participants, and between stimuli presented at 4 ° and 6 ° eccentricity. Viewing distances were estimated and controlled for by a ‘virtual chinrest’ which measured the visual angle between participants’ central fixation and blind spot. Agreement between web- and lab-based measurements in our spatial 2AFC task, which presented stimuli at 8 ° eccentricity, supports the use of stimuli in the near-periphery in web-based visual crowding tasks, although this remains to be demonstrated in clinical populations.

Remote, web-based tracking of visual function can also provide insight that would be impractical to reveal with traditional lab-based methods. For example, Shepherd (2020) had subjects with migraine regularly perform a web-based global motion coherence task and found that thresholds for discriminating vertical motion were significantly elevated two days pre- and post-migraine attack. Evaluations of the agreement between web- and lab-based measurements should still be made if global motion tasks are to be used in assessments of visual dysfunction. Nonetheless, if performance on a web-based task has predictive value of patient symptomology (Shepherd, 2020), then it may still be useful as a biomarker of active or ongoing cortical disruption.

Finally, in some settings, it is useful to collect behavioural performance data alongside self-reported measures of disability (at an individual or population level). Impaired function, as opposed to the presence or severity of ocular disease, often provides better insight into the problems patients experience with daily activities (Ivers et al., 2000). In situations where visual function is deteriorating relatively rapidly, performance loss and its impact on visual quality of life could be monitored in tandem via web-based interfaces.

## Conclusions

We found a statistically robust oblique effect in an orientation-identification task performed online and have demonstrated strong agreement between web- and lab-based measurements. Although explorations of group differences and task-based effects may still prove useful, we recommend that future web-based research use forced-choice designs along with a validation-subsample, to compare objective performance on web- and lab-based versions of a task.

## Data Availability

The datasets generated and analysed during the current study are available from https://github.com/RichardJamesLeadbeater/Web_vs_Lab
